# Thyroid diseases in pregnancy: The importance of anamnesis

**Published:** 2013

**Authors:** Necati Bulmus, Isik Ustuner, Emine Seda Guvendag Guven, Figen Kir Sahin, Senol Senturk, Serap Baydur Sahin

**Affiliations:** 1 Necati Bulmus, Departments of Family Medicine, Faculty of Medicine, Recep Tayyip Erdogan University, Rize, Turkey.; 2 Isik Ustuner, Departments of Obstetrics andGynecology, Faculty of Medicine, Recep Tayyip Erdogan University, Rize, Turkey.; 3 Emine Seda Guvendag Guven, Departments of Obstetrics andGynecology, Faculty of Medicine, Recep Tayyip Erdogan University, Rize, Turkey.; 4 Figen KirSahin, Departments of Obstetrics andGynecology, Faculty of Medicine, Recep Tayyip Erdogan University, Rize, Turkey.; 5 Senol Senturk, Faculty of Medicine, Recep Tayyip Erdogan University, Rize, Turkey.; 6 Serap Baydur Sahin, Departments of Endocrinology, Faculty of Medicine, Recep Tayyip Erdogan University, Rize, Turkey.

**Keywords:** Hyperthyroidism, Hypothyroidism, Thyroid diseases in pregnancy, Goiter

## Abstract

***Objective:*** Primary objective of our study was to evaluate the efficiency of detailed medical history and thyroid examination of the pregnant women presenting to our clinic from Rize province and nearby which was an endemic goiter region. It was aimed to investigate the frequency of thyroid diseases, pregnancy outcomes and the efficiency of screening with thyroid function tests during the first trimester of pregnancy as secondary endpoint.

***Methodology*** ***:*** A prospective clinical study was conducted with 998 pregnant women between the ages of 17-48 years. In the first step of our study, a detailed medical history was obtained and a detailed thyroid gland examination was performed in all the patients (n=998). In the patients diagnosed with thyroid disease or considered to have thyroid disease with these results (n=107), thyroid diseases were evaluated with thyroid function tests and imagining methods. Analyses of socio-demographic data and nutrition were also made. In the second step, thyroid stimulating hormone (TSH), free T3 and free T4 tests were performed in the first antenatal examination of the pregnant cases considered not to have thyroid disease after medical history and examination (n=891). Parameters of thyroid peroxidase antibodies (TPOAb), thyroglobulin antibodies (TgAb) and TSH receptor auto antibodies (TRAb) were investigated in the cases whose TSH, sT3 and sT4 levels were different than the reference values after examination of the endocrinologist. Thyroid ultrasonography was performed. Urinary iodine levels in 24 hour urine were investigated.

***Results: ***During pregnancy, the incidence of hyperthyroidism and hypothyroidism in the whole study group were 2.8% (28/998) and 4.3% (43/998), respectively, 6.7% of the patients (67/998) had a diagnosis of thyroid disease before pregnancy. Hyperthyroidism and hypothyroidism depending on the TSH screening results were 1.9% (17/891) and 1.1% (10/891) respectively and the incidence of overt hyperthyroidism and overt hypothyroidism were 0.2% (2/891) and 0.2% (2/891) in the pregnant cases considered not to have thyroid disease with medical history and examination.

***Conclusion: ***Detailed medical history and family history obtained during the first trimester of pregnancy helped us to identify 6.7% of thyroid diseases among the pregnant women. This result effectively emphasizes the importance of detailed first prenatal examination regarding the thyroid.

## INTRODUCTION

Many changes occur in the thyroid gland, thyroid functions, iodine metabolism and immune system during pregnancy.^1^ Physiological changes of the pregnancy may mimic the thyroid disease and pregnancy may lead to changes in the clinical condition of the thyroid disease. Pregnancy period is a stressful period both for expectant mother and the physician regarding the diseases and the drugs used. Therefore, not knowing the changes occurring during the pregnancy in thyroid diseases and the treatment of thyroid diseases may cause this period to result in harmful effects for the mother and the fetus. Regarding correct diagnosis and treatment of thyroid diseases in pregnancy, knowing the changes occurring in thyroid physiology and thyroid function tests is important for prevention of maternal and fetal morbidity.

Primary objective of our study was to evaluate the efficiency of detailed medical history and thyroid examination of the pregnant women presenting to our clinic from Rize province and nearby which was an endemic goiter region. It was also aimed to investigate the frequency of thyroid diseases, pregnancy outcomes and the efficiency of screening with thyroid function tests during the first trimester of pregnancy as secondary endpoint.

## METHODOLOGY

This prospective study was approved by the Institutional Ethical Committee of the institution in which this study was conducted. Written consent for participation was obtained after the design and aim of the study was explained to all participants. A prospective, two-step and single-center clinical study was conducted.The sample size was calculated using the Number Cruncher (NCSS) Power Analysis and Sample Size (PASS) program. The prevalence of thyroid disease ratios in pregnancy were taken into account during the calculation as well as the error margins and confidence intervals thus, 998 patients between 17-48 years of age from April 2011 to January 2012 were included in this study. 

Antenatal care was provided as per hospital protocol. The routine pregnancy follow-up visit was started in the first trimester(<14^th^ week) in all the pregnant women. In the first step of our study, a detailed medical history was obtained and a general physical examination was performed in all the patients (Group I) (n=998). As a part of physical examination, thyroid gland was evaluated by palpation and it was classified as grade 0, 1 and 2.^2^All laboratory and imagining tests performed regarding the thyroid gland were recorded. In the cases diagnosed with thyroid disease or considered to have thyroid disease with this results (n=107), parameters of thyroid stimulating hormone (TSH), free T3, free T4, thyroid peroxidase antibodies (TPOAb), thyroglobulin antibodies (TgAb) and TSH receptor auto antibodies (TRAb) were investigated after examination of the endocrinologist. Thyroid ultrasonography was performed. Urinary iodine levels in 24 hr urine were investigated. Chemiluminescent enzyme immunometric assay was used in determination of thyroid function tests (Abbott Architect I 2000 SR, Abbott).

**In the second step of our study,** thyroid stimulating hormone (TSH), free T3 and free T4 tests were performed in the first antenatal examination of the pregnant cases considered not to have thyroid disease with medical history, examination and previous laboratory and imagining results (Group II) (n=891). Parameters of thyroid peroxidase antibodies (TPOAb), thyroglobulin antibodies (TgAb) and TSH receptor auto antibodies (TRAb) were investigated in the cases whose TSH, sT3 and sT4 levels were different than the reference values after examination of the endocrinologist. Thyroid ultrasonography was performed. Urinary iodine levels in 24 hr urine were investigated. 

Normal reference values were as followings: TSH =0.1-2.5 mU/L, free T3= 2.5-3.9pg/mL, free T4= 0.8-2.0 ng/mL, Anti-TPO= 0-34 IU/mL, Anti-Tg= 0-115 IU/mL. For the diagnosis of Hashimoto thyroiditis in the cases determined as having hypothyroidism, the following criteria were considered to be present: heterogeneous pattern in thyroid USG, pseudonodules and/or diffuse bands together with Anti-TPO and/or Anti-Tg positivity.

The patients were diagnosed with overt hyperthyroidism, overt hypothyroidism, subclinical hyperthyroidism and subclinical hypothyroidism by evaluating these results.

Medical, reproductive data and socio-demographic characteristics of the cases were recorded on the patient follow-up visit forms with face-to-face interview. On the patient follow-up visit form: age, gravida, parity, the number of abortion, weight and height data, socioeconomic status, smoking, alcohol consumption, any previous treatment related to thyroid diseases, any previous surgical treatment related to thyroid gland, presence of family history of thyroid disease and Type 1 diabetes mellitus and autoimmune disease like rheumatoid arthritis were recorded. The pregnant women with thyroid disease were followed up together with an endocrinologist.

Obstetrical and neonatal outcomes of the cases were evaluated by obtaining records from follow-up visit and delivery files and newborn unit files. Gestational age at delivery, birth weight, preeclampsia, preterm delivery, intrauterine growth retardation and gestational diabetes rates were determined. Hyperthyroidism and hypothyroidism findings in the fetus and newborns were evaluated. Thyroid hormone replacement therapy or changes in the doses of antithyroid medications during antenatal follow-up visits were investigated.

***Statistical analysis: ***Statistical analysis was performed by using SPSS (Statistical Package for Social Sciences) software package version 11.5. During the evaluation of the study data, regarding the comparisons of descriptive statistical methods (Mean, Standard deviation, frequency) as well as quantitative data, Kruskal Wallis Test was used for the intergroup comparisons of parameters without normal distribution. Student-t test was used for the intergroup comparisons of parameters with normal distribution and Mann Whitney U test was used for the intergroup comparisons of parameters without normal distribution. Chi-Square test and Fisher’s Exact Chi-Square test were used for comparison of qualitative data. The significance of the results was evaluated in 95% confidence interval and p<0.05 was considered to be statistically significant.

**Table-I:** Clinical characteristics of hypothyroidism cases in Group I (n=33).

*Etiology*	
Hashimoto thyroiditis	8 (24.2%)
Iodine deficiency	11 (33.3%)
Surgical thyroidectomy	14 (42.4%)
Duration of hypothyroidy (year)	6.5 ± 4.8
Age	33.3 ± 4.9
Rate of abortion history	8/33 (24.2%)
Rate of habitual abortion	2/33 (6.0%)
Average birth weight (g)	3108 ± 548
Average birth week	38.2 ± 2.7
Pregnancy complications	
Intrauterine growth retardation	1/33 (3.0%)
Preterm delivery (< 37 weeks)	3/33 (9.0%)
Preeclampsia	1/33 (3.0%)
Gestational diabetes	3/33 (9.0%)

**Table-II:** Clinical characteristics of hyperthyroidism cases in Group I (n=11).

*Etiology*	
Graves’ disease	9 (81.8%)
Toxic multinodular goiter	2 (18.1%)
Duration of hyperthyroidy (year)	4.1 ± 3.8
Age	32.3 ± 3.9
Rate of abortion history	2/11 (18.1%)
Rate of habitual abortion	-
Average birth weight (g)	3056 ± 518
Average birth week	38.1 ± 2.6
Pregnancy complications	
Intrauterine growth retardation	1/11 (9.0%)
Preterm delivery (< 37 weeks)	1/11 (9.0%)
Preeclampsia	1/11 (9.0%)
Gestational diabetes	1/11 (9.0%)

**Table-III:** Medical and reproductive characteristics of the pregnant women (Group II) (n=891).

*Characteristics*	*X ± S. deviation, median (min-max)*
Age (year)	28.5 ± 5.5, 28 (17-48)
Gravida	2.1 ± 1.3, 2 (1-11)
Parity	0.9 ± 0.9, 1 (0-6)
Abortion	0.2 ± 0.6, 0.00 (0-6)
Alive	0.9 ± 0.9, 1.00 (0-5)
Average birth week	38.9 ± 1.8, 39 (16-41)
Average birth weight (g)	3321.7 ± 480.1
Pregnancy complications	
Intrauterine growth retardation	11/891 (1.2%)
Preterm delivery (< 37 weeks)	49/891 (5.5%)
Preeclampsia	28/891 (3.1%)
Gestational DM	35/891 (3.9%)

**Table-IV:** Socio-demographic characteristics of the pregnant women (Group II) (n=891).

*Characteristics*		*n(%)*
Smoking during pregnancy	Yes	24 (2.7%)
	No	837 (93.9%)
	Quitted	30 (3.4%)
Maternal education level	High	148 (16.6%)
	Intermediate	296 (33.2%)
	Low	447 (50.2%)
Occupation	Housewife	740 (83.1%)
	Working	151 (16.9%)
Socioeconomic status	High	115 (12.9%)
	Intermediate	689 (77.3%)
	Low	87 (9.8%)
Multivitamin use during pregnancy	Yes	411 (46.1%)
	No	480 (53.9%)
Nutrition level during pregnancy	Good	774 (86.9%)
	Poor	117 (13.1%)

## RESULTS

In the first antenatal examination (<14^th^week), presence of thyroid disease was determined from the detailed medical history in 107 cases in Group I. Thirty-three, 11 and 23 of these pregnant women in the first step of our study were the cases that were being followed-up due to hypothyroidism, hyperthyroidism and diffuse or nodular goiter, respectively. Eight (24.2%), 11 (33.3%) and 14 (42.4%) of hypothyroidism cases were the hypothyroidism patients developed due to Hashimoto thyroiditis, iodine deficiency and thyroidectomy performed for thyroid nodule or hyperplasia, respectively (Table-I).

All these cases were the patients who were diagnosed before the pregnancy and in whom L-thyroxine treatment was started. Average TSH levels at presentation were 2.1mU/L (levels within the last six months before the pregnancy were taken into consideration). Hypothyroidy symptoms were not developed in the pregnant women during antenatal follow-up visits. In 31 (93.9%) of the pregnant women, the dosages of L-thyroxine used before the pregnancy were increased during follow-up visits. The dosage was increased in all of the cases with Hashimo to thyroiditis and in all of the cases under going thyroidectomy after surgery. The dosage was also increased in 9 (9/11, 81.8%) of caseswithiodinedeficiency. No problem was observed in the newborns of the hypothyroidy cases.

When the hyperthyroidy cases were investigated; 9 (81.8%) cases were determined to be Graves’ disease and 2 cases (18.1%) to be hyperthyroidy due to toxic multi nodular goiter (Table-II).

Intrauterine growth retardation and preeclampsia developed in a case with Graves’ disease. The baby delivered at a gestational age of 34 weeks by emergency cesarean section was discharged without developing any complication following a 3-day hospitalization in neonatal unit. Antithyroid medication was not used in three cases with Graves’ disease. However, antithyroid medication was used in six cases during pregnancy. Thyroid functions were kept under control in all of the cases during pregnancy as free T4 level to be at upper limit of normal range. Goiter or other fetal hypothyroidy findings were not observed at the postpartum examinations of the newborns of the pregnant women used antithyroid medication during follow-up visits.

When the cases diagnosed with diffuse or nodular goiter before the pregnancy (n=23) were investigated; it was observed that 14 cases were diagnosed with euthyroid diffuse goiter and iodine was added to their diets and 10 of them used L-thyroxine treatment for a while. Etiology was iodine deficiency in all of the cases. None of the cases were using treatment in the first antenatal examination (<14^th^ week). No pregnancy complication was observed in the cases.

While there was solitary nodule in 6 of the cases diagnosed with nodular goiter (n=9), multiple nodules were determined in 3 of them. During the investigation of the medical histories obtained from the patients and the test results, all of the nodules in the cases with solitary nodule were >10 mm. In nuclear medical examinations (scintigraphies) of these patients performed before the pregnancy (n=6), cold thyroid nodule was determined in 4 cases, hot thyroid nodule was determined in one case and warm thyroid nodule was determined in 1 case. In thyroid USG examinations was performed, nodules were observed to be hypoechoic pattern in 4 cases, cystic pattern in one case and solid pattern in one case. However, no malignancy was determined in the results of fine-needle aspiration biopsies of the cases (n=6).

Two or more nodules were determined in thyroid USG examinations of 2 of the cases diagnosed with multinodular goiter (n=3), but no malignancy was determined in the results of fine-needle aspiration biopsies of the cases. Thyroid scintigraphy was performed for the evaluation of newly detected multinodulargoiter in one case that was unaware of her pregnancy and the case presented for the first antenatal examination. While the case was in the evaluation step, she had medically-induced abortion by dilation and curettage in an outer center. No pregnancy complication was observed in the cases diagnosed with nodular goiter.

In the first antenatal examination, by thyroid gland palpation, there was grade 1 goiter in 36 cases and grade 2 goiter in 4 cases of the remaining patients except the aforementioned ones. All of grade 1 and grade 2 cases (n=40) were euthyroid. All of the cases diagnosed with grade 1 goiter were diagnosed as euthyroid diffuse goiter after clinical evaluation and thyroid USG performed. Multinodular goiter was determined in 2 of 4 cases determined to have grade 2 goiter after clinical evaluation and thyroid USG performed and fine-needle aspiration biopsies were performed in the nodules suspected for malignancy. Results of the cases were evaluated to be benign. No pregnancy complication was observed in the cases whose diets were regulated and who were followed-up with thyroid function tests.

In the first antenatal examination (<14^th^ week), thyroid disease was not determined to be present from the medical history in 891 cases(Group II). Average age of the cases (n=891) in the second step of our study was 28.5±5.5 years and average birth week was 38.9 ± 1.8 weeks. All the pregnant women participating in our study had a regular antenatal follow-up visit and 311 cases (34,9%) were primigravid and 580 cases (65,1%) were multiparae (Table-III).Socio-demographic characteristics of the pregnant women participating in our study shown in Table-IV.

When the first trimester TSH levels of the pregnant women were investigated; TSH levels were measured in 891 cases and it was determined to be below the normal values (< 0.1 mU/L) in 17 cases (2.1%) and to be above the normal values (> 2.5 mU/L) in 10 cases (1,2%). Average TSH level in the study group was found to be 1.03 ± 1.10, 1.40 (0.01-14.87) mU/L. When the cases diagnosed during pregnancy were investigated, there was subclinical hyperthyroidism due to hCG-dependent hyperthyroidism in 15 cases with thyrotoxicosis and hyperthyroidism due to Graves’ disease in 2 cases. There was subclinical hypothyroidism due to iodine deficiency in 8 of 10 cases determined to have hypothyroidy. Hypothyroidy due to Hashimoto thyroiditis was determined in two cases.

Hyperthyroidy frequency in the pregnant population in which TSH screening was performed in the second step of our study was found to be 1.9% (17/891) and the incidence of Graves’ disease diagnosed with screening during pregnancy to be 0.2% (2/891). However, when all the first and second step study groups are evaluated, the frequency of hyperthyroidy during pregnancy in our hospital is 2.8% (28/998).

Hypothyroidy frequency in the pregnant population in which TSH screening was performed in the second step of our study was found to be 1.1% (10/891) and the incidence of hypothyroidy diagnosed with screening during pregnancy to be 0.2% (2/891). However, when all the first and second step study groups are evaluated, the frequency of hypothyroidy during pregnancy in our hospital was 4.3% (43/998).

## DISCUSSION

Thyroid diseases are endocrine disorders which may lead to deleterious outcomes for the mother and fetus during pregnancy. Therefore, during medical monitoring of the pregnant woman with thyroid disease, primary care physician should follow up the pregnancy in cooperation with gynecologist and endocrinologist with a multidisciplinary approach.

Increase in deleterious pregnancy outcomes and neurodevelopmental problems in the newborn together with thyroid dysfunction during pregnancy in the observational studies performed made routine screening of thyroid diseases in early pregnancy a current issue. Although there is a consensus on screening of high-risk group, universal screening is not recommended yet.^3^^-^^6^ Additionally, studies are also needed regarding best screening criterion, most appropriate time for the screening and the efficiency of the treatment that will be used in the cases with positive screening result.

If the overt thyroid disease is not diagnosed and treated during pregnancy, it leads to severe maternal and fetal complications. If the overt thyroid disease is present in the first trimester of the pregnancy, these deleterious outcomes are much more.^7^ The data about the effects of subclinical hypothyroidy and hypothyroxiemia is scarce. In the observational studies performed, delay in meaningful speech and in expressive language, retardation in mental and motor functions, low IQ and poor neonatal adaptation were shown in the children of the pregnant women with hypothyroxiemia in early pregnancy.^8^^-^^11^ However, in other studies performed, no correlation was demonstrated between subclinical thyroid dysfunction and cognitive and behavioral problems of the child.^12^ It was reported that thyroid autoimmunity has also deleterious effects in the pregnancy independently from maternal thyroid function.

During the first prenatal examination in the first step of our study, we investigated the thyroid functions with detailed medical history. Six point seven percent (6.7/998) of the cases in our study group were the patients who were diagnosed, followed-up and treated before the pregnancy. This result can be explained as being endemic goiter region of Rize province and East Black Sea region and reference hospital characteristic of our hospital. Additionally, it is the indicator of favorable outcomes of public health screening programs performed since our region is an endemic goiter region.

Hyperthyroidy and hypothyroidy frequencies in the pregnant population in which TSH screening was performed in the second step of our study were found to be 1.9% (17/891) and 1.1% (10/891), respectively. Incidences of overt hyperthyroidy and overt hypothyroidy were determined to be 0.2% (2/891) and 0.2% (2/891), respectively. In the literature, general hyperthyroidy frequency in all of the pregnancies was shown to be 0.1-0.4% and hypothyroidy frequency in the pregnant women screened to be 0.3-0.5%.^5^^,^^13^^-^^16^ When the entire study group was evaluated, the incidence of hyperthyroidy and hypothyroidy during pregnancy in our hospital were 2.8% (28/998) and 4.3% (43/998), respectively.

Today, two large randomized studies evaluate the efficiency of screening in the early pregnancy. In their study conducted in 4562 pregnant women living in an iodine-deficiency region, Negro et al compared the universal screening and screening for the target cases.^17^The rate of the patients at high-risk in the screening group is 21% in this study. This means that while all of five patients presenting for the pregnancy will be included in thyroid screening when universal screening is performed; while only one of the five patients is screened, the remaining patients will not be screened when patients at high-risk are targeted. In the group in which universal screening was performed, euthyroid, hypothyroid and hyperthyroid cases were determined to be at the rates of 96.8%, 2.8% and 0.4%, respectively. As a conclusion, it could not be shown that universal screening decreased the obstetric and neonatal complications compared to the group screened the target cases. Investigators noted down that only one pregnant woman would need treatment when 36 pregnant women were screened in low-risk cases and deleterious pregnancy outcomes could be prevented in only one pregnant woman with the treatment when 60 pregnant women were screened in low-risk cases. 

Lazarus et al evaluated 21846 pregnant women and their children in their large-scale randomized studies.^18^ It showed that significant increase did not occur in the cognitive functions in three years of age with the treatment.

The most important finding in our study is determination of thyroid disease by us in 6.7% of the pregnant women with detailed medical history and family history obtained in the first trimester of the pregnancy in our region which is an endemic region. This result emphasizes the importance of medical history and thyroid examination performed effectively in the first prenatal examination.

In conclusion, overt hyperthyroidy (n=2, 0.2%) and overt hypothyroidy cases (n=2, 0.2%) were determined with the screening in the first prenatal visit. These cases would lose their chances for diagnosis and therapy if the screening was not performed.

Our study has some methodological limitations. The scale of the study group should be larger to compare the socio-demographic characteristics and the rates of obstetric and neonatal complications of the cases of overt thyroid dysfunction determined with the screening with the other groups. Also selection of the study group from a single center (reference hospital) resulted in a biased selection and sampling. However, we believe that our study will enlighten the other studies that will be performed in this field with regards to its findings.
